# Computational Prediction of Protein-Protein Interactions of Human Tyrosinase

**DOI:** 10.1155/2012/192867

**Published:** 2012-03-26

**Authors:** Su-Fang Wang, Sangho Oh, Yue-Xiu Si, Zhi-Jiang Wang, Hong-Yan Han, Jinhyuk Lee, Guo-Ying Qian

**Affiliations:** ^1^College of Biological and Environmental Sciences, Zhejiang Wanli University, Ningbo 315100, China; ^2^Korean Bioinformation Center (KOBIC), Korea Research Institute of Bioscience and Biotechnology, Daejeon 305-806, Republic of Korea; ^3^Department of Biology, College of Life Sciences, Soochow University, Suzhou 215123, China; ^4^Department of Bioinformatics, University of Sciences and Technology, Daejeon 305-350, Republic of Korea

## Abstract

The various studies on tyrosinase have recently gained the attention of researchers due to their potential application values and the biological functions. In this study, we predicted the 3D structure of human tyrosinase and simulated the protein-protein interactions between tyrosinase and three binding partners, four and half LIM domains 2 (FHL2), cytochrome b-245 alpha polypeptide (CYBA), and RNA-binding motif protein 9 (RBM9). Our interaction simulations showed significant binding energy scores of −595.3 kcal/mol for FHL2, −859.1 kcal/mol for CYBA, and −821.3 kcal/mol for RBM9. We also investigated the residues of each protein facing toward the predicted site of interaction with tyrosinase. Our computational predictions will be useful for elucidating the protein-protein interactions of tyrosinase and studying its binding mechanisms.

## 1. Introduction

Tyrosinase (EC 1.14.18.1) is ubiquitously distributed in organisms and is a critical enzyme involved in melanin production, with multiple catalytic functions in pigment production [1–3]. Tyrosinase mutations are directly linked to pigmentation disorders in mammals [4, 5] and can cause a browning effect in vegetables [6, 7]. In addition, tyrosinase participates in cuticle formation in insects [8, 9]. In mammals, tyrosinase is a bifunctional enzyme that first converts tyrosine to DOPA and then to DOPA quinone, which is further cyclized and oxidized to produce melanin pigments [10]. The human tyrosinase protein contains two Cu^2+^-binding sites, two cysteine rich regions, a signal peptide region, a transmembrane anchor domain, and an EGF motif [11]. Two Cu^2+^ ions in the active site of tyrosinase are coordinated by three histidine residues each and are essential for the enzyme's catalytic activity [12]. Furthermore, the presence of Cu^2+^ in the active site of tyrosinase is observed across numerous organisms [13]. Therefore, chelation of tyrosinase Cu^2+^ by synthetic compounds or agents from natural sources has been targeted as a way to block tyrosinase catalysis for medicinal purposes, darkening problems in agricultural products, and cosmetic interests [14, 15].

As the crystallographic structure of tyrosinase has been gradually elucidated, insights into its catalytic mechanisms and active site have also been revealed [16–18]. However, while the catalytic mechanism of tyrosinase-mediated melanin pigment production has been well studied, the relationship between tyrosinase enzyme activity and protein interactions has not been fully elucidated, despite several reports of interacting proteins for tyrosinase [19–22].

Loss of tyrosinase activity causes oculocutaneous albinism type 1 (OCA 1) in humans [23]. Specifically, studies have identified over 100 different missense, nonsense, insertion, or deletion nucleotide mutations dispersed rather evenly over the entire tyrosinase gene [24].

In the present study, we modeled the 3D structure of tyrosinase and simulated its protein-protein interactions to understand the structural mechanisms of the binding between tyrosinase and its partners. We deduced the binding sites between tyrosinase and three known interaction partners, four and half LIM domains 2 (FHL2), cytochrome b-245 alpha polypeptide (CYBA), and RNA-binding motif protein 9 (RBM9) and describe their potential regulatory effects with respect to substrate accessibility at the active site of tyrosinase.

## 2. Materials and Methods

### 2.1. 3D Structure Homology Modeling of Human Tyrosinase

A three-dimensional model of tyrosinase consisting of 377 aminoacids was constructed using SWISS-MODEL [25, 26] based on homology modeling. The program automatically provides an all-atom model using alignments between the query sequence and known homologous structures. We retrieved known homologous structures of tyrosinase from the Protein Data Bank (PDB) (http://www.pdb.org/) and identified a partially homologous protein (PDB entry: 3NM8 chain B, 32% sequence identity) to serve as a structural template for tyrosinase. Based on the sequence alignment, the 3D structure of tyrosinase was constructed with a high level of confidence.

### 2.2. Homology Modeling of 3D Structures for FHL2, RBM9, and CYBA

Using the same method described for modeling the structure of tyrosinase, we retrieved known homologous structures from the PDB as follows: 1X4L chain 1 for FHL2 and 2CQ3 chain 1 for RBM9. Structural template sequence identities for FHL2 and RBM9 were 100% and 98%, respectively. In the case of CYBA, there was an available 3D structure in the PDB as 1WLP chain A (identity 100%).

### 2.3. In Silico Protein-Protein Interactions between Tyrosinase and FHL2, RBM9, and CYBA

There are many tools available for *in silico* protein-protein docking. In the present study, we used the HEX program [27] because of its success in the CAPRI (Critical Assessment of Predicted Interactions; http://capri.ebi.ac.uk/) competition with respect to proposing good docking solutions. HEX determines the steric shape, electrostatic potential, and charge density of each protein as expansions of spherical polar Fourier basis functions. The protein surface shapes are calculated to determine the match potential of two proteins. Then, candidate-docking solutions are refined using a “soft” molecular mechanics energy minimization procedure, and the list of docking solutions is clustered to assist in identifying distinct orientations.

## 3. Results and Discussion

### 3.1. Computational Prediction of 3D Tyrosinase Structure

**Figure 1:**
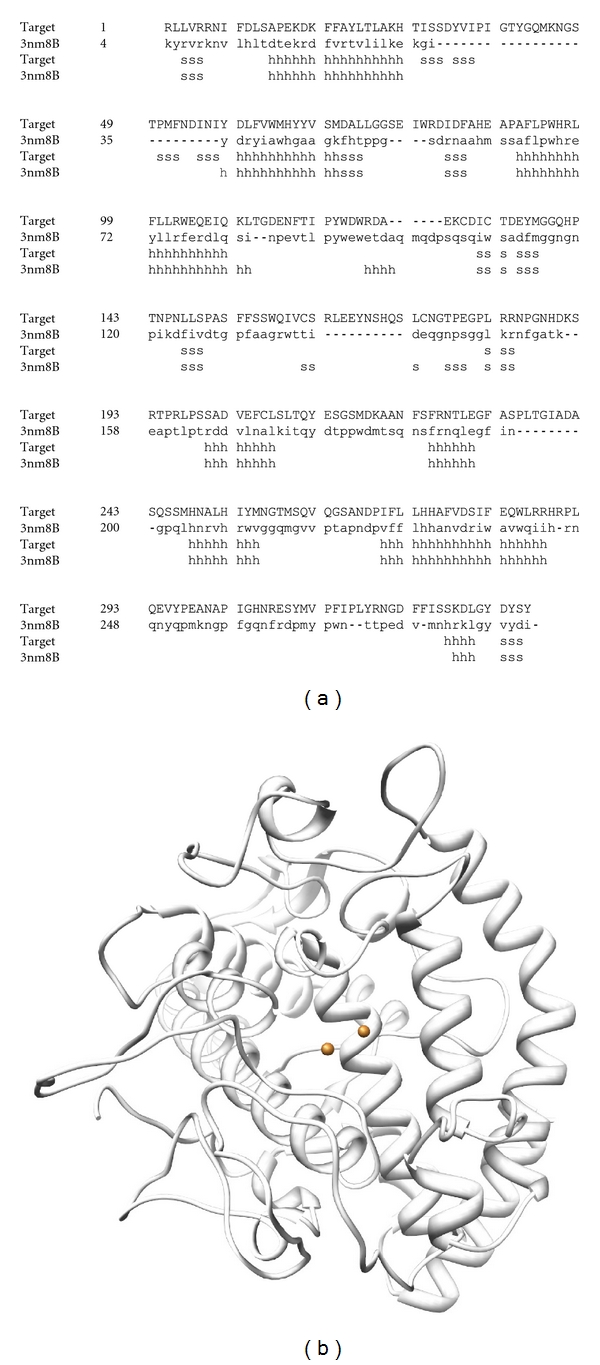
(a) Alignment of the human tyrosinase target and template structure (3NM8). (b) Illustration of the predicted tyrosinase structure modeled by SWISS-MODEL; the spheres represent Cu^2+^.

The accuracy of structure prediction during homology modeling depends strongly on sequence identity between a query sequence and template structures. In order to simulate tyrosinase 3D structure, we selected a template structure from PDB entry as 3NM8 chain B. The sequence identity was 32%, as shown in Figure 1(a). In the predicted structure of tyrosinase, the binding pocket was located close to two Cu^2+^ ions (Figure 1(b)). Since the crystallographic structure of human tyrosinase has not been elucidated, it is unclear which residues are glycosylated in the tyrosinase structure. A previous report revealed that human tyrosinase was highly glycosylated [28–30], and it is associated with the correct folding to form the active enzyme. The Cu^2+^-binding site exists in the tyrosinase active site pocket, and it will be interesting to further study the role of Cu^2+^ on the conformation stability in addition to the catalytic role.

### 3.2. Computational Predictions of 3D Structures of FHL2, RBM9, and CYBA

**Figure 2:**
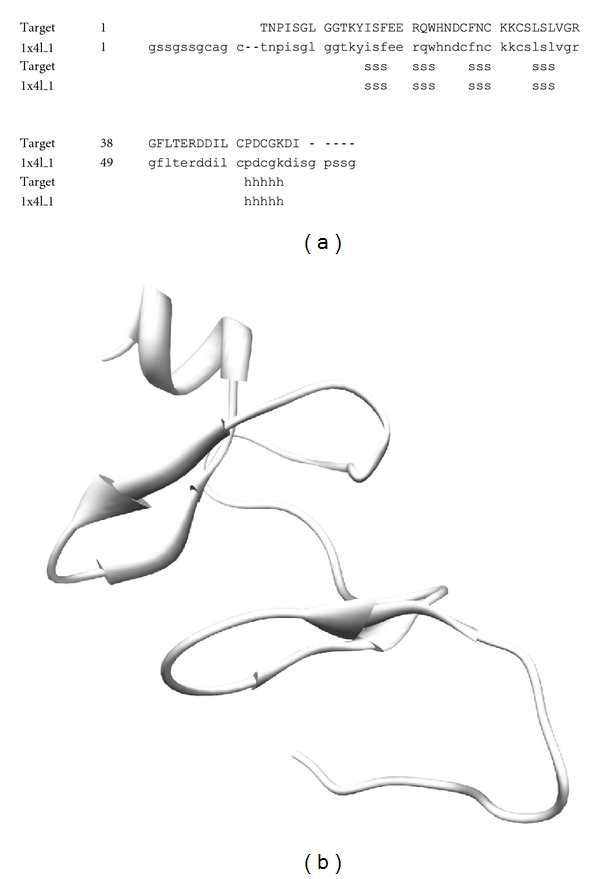
(a) Alignment of the FHL2 target and its template structure (1X4L). (b) Illustration of the predicted target structure modeled by SWISS-MODEL.

**Figure 3:**
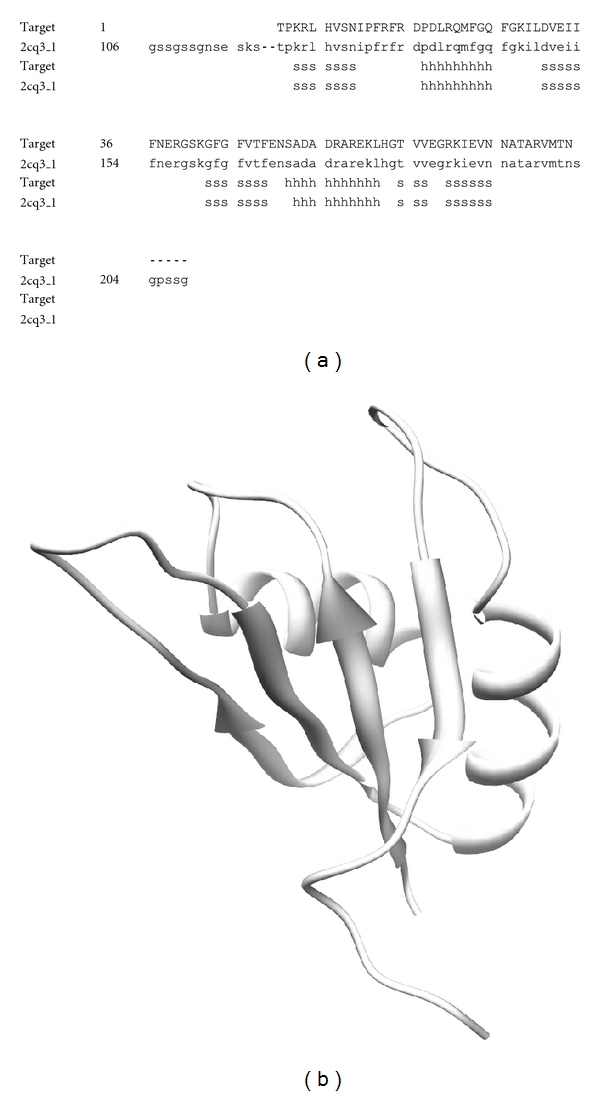
(a) Alignment of the RBM9 target and its template structure (2CQ3). (b) Illustration of the predicted target structure modeled by SWISS-MODEL.

**Figure 4:**
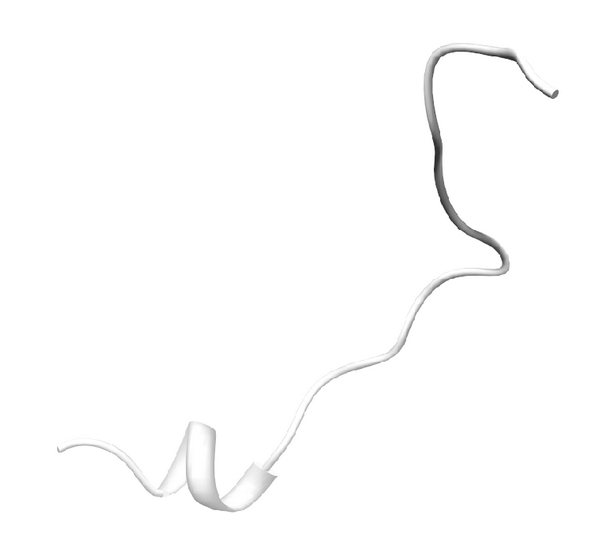
Predicted CYBA structure modeled by SWISS-MODEL based on a template structure (PDB ID: 1WLP).

The 3D structure of FHL2 was constructed with 100% sequence identity compared to the template 1X4L chain 1 (Figure 2). In the same way, RBM9 was also constructed with 98% sequence identity compared to the template 2CQ3 chain 1 (Figure 3(a)). As a result of alignment, RBM9 was predicted to contain two helical structures and four beta sheet structures (Figure 3(b)). Meanwhile, the 3D structure of CYBA was modeled with 100% sequence identity template structure (1WLP chain A) (Figure 4).

### 3.3. Docking Simulation between Tyrosinase and FHL2

**Figure 5:**
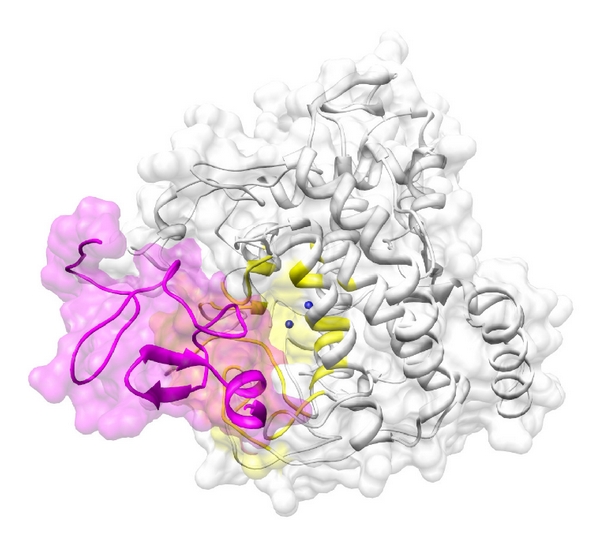
Docking between tyrosinase (white) and FHL2 (magenta). The active site of tyrosinase near two the Cu^2+^ ions (blue spheres) is colored in yellow. The two proteins are depicted as illustrations.

The docking between tyrosinase and FHL2 was successful, with significant scores (Eshape score: −543.4 kcal/mol; Eforce score: −51.9 kcal/mol; total score: −595.3 kcal/mol) shown in Figure 5. When searching for binding residues on the surface of tyrosinase facing toward FHL2, we detected LEU58, PHE60, CYS78, THR79, HIS80, GLY81, ASP173, PRO174, SER175, PHE176, LYS177, PRO178, TYR179, GLY180, ASP181, PHE182, ALA183, TRP185, HIS234, GLY235, ILE236, SER237, ASP238, ASP239, GLN240, VAL254, TYR258, LYS260, ILE261, GLU262, ASP266, HIS267, PRO268, PHE269, PHE270, ARG306, ASP307, and GLY308. For FLH2, we found that ASN2, PRO3, ILE4, SER5, GLY6, THR10, LYS11, TYR12, ILE13, TRP20, HIS21, ASN22, ASP23, CYS24, PHE25, ASN26, LYS29, CYS30, SER31, LEU32, SER33, LEU34, VAL35, GLY36, ARG37, GLY38, CYS48, PRO49, ASP50, CYS51, LYS53, and ASP54 were important for the interaction with tyrosinase. Interestingly, several of these residues are known to interact with some inhibitors of tyrosinase [31–35] and are located near the binding sites of FHL2, suggesting that FHL2 may alter the activity of tyrosinase during catalysis.

### 3.4. Docking Simulation between Tyrosinase and RBM9

**Figure 6:**
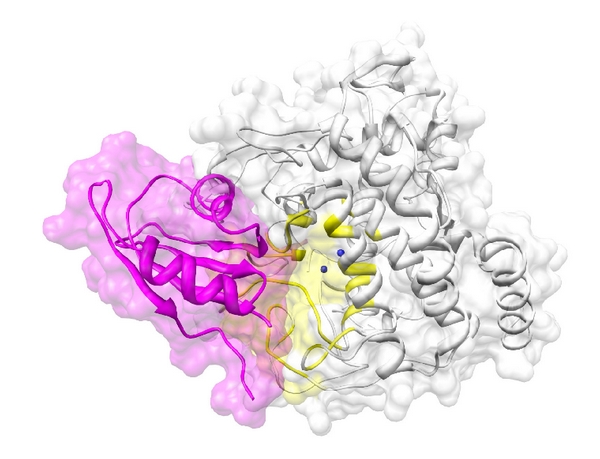
The docking between tyrosinase (white) and RBM9 (magenta). The active site of tyrosinase near two the Cu^2+^ ions (blue spheres) is colored in yellow. The two proteins are depicted as illustrations.

As with the case of FHL2, the docking between tyrosinase and RBM9 was successful with significant scores (Eshape score: −609.8 kcal/mol; Eforce score: −211.5 kcal/mol; total score: −821.3 kcal/mol), as shown in Figure 6. When searching for binding residues on the surface of tyrosinase facing toward RBM9, we detected LEU58, PHE60, LYS74, ALA75, GLY76, ILE172, PRO174, SER175, PHE176, LYS177, PRO178, TYR179, GLY180, ASP181, PHE182, ALA183, THR184, TRP185, ARG186, THR187, ARG194, ASN195, ARG196, ARG197, HIS234, GLY235, ILE236, SER237, ASP238, ASP250, GLU262, GLY263, HIS264, ASP266, HIS267, PRO268, PHE269, PHE270, ARG306, ASP307, and GLY308. For RBM9, numerous residues including THR1, PRO2, ARG4, VAL7, SER8, ASN9, ILE10, PRO11, PHE12, ARG13, PHE14, ARG15, ASP16, PRO17, ASP18, LEU19, ARG20, GLN21, MET22, PHE23, GLY24, GLN25, GLY27, LYS28, ILE29, LEU30, ASP31, VAL32, GLU33, ILE34, PHE36, GLY43, PHE44, GLY45, PHE46, VAL47, THR48, GLU50, ILE72, ARG80, VAL81, MET82, and ASN84 were predicted to interact with tyrosinase. By comparing these results with those of FHL2, we found that most of the predicted residues on tyrosinase were common with that of RBM9, implying that the regulatory effect of RBM9 on the activity of tyrosinase might be similar to that of FHL2, as they both dock close to the active site of tyrosinase.

### 3.5. Docking Simulation Between Tyrosinase and CYBA

**Figure 7:**
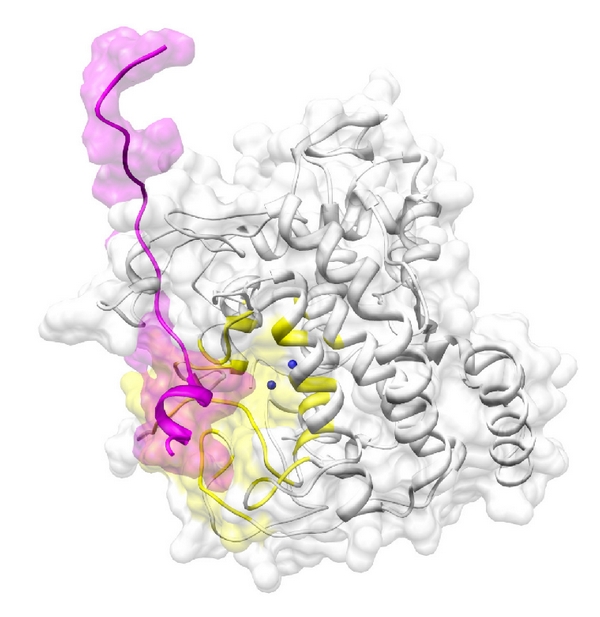
The docking between tyrosinase (white) and CYBA (magenta). The active site of tyrosinase near two the Cu^2+^ ions (blue spheres) is colored in yellow. The two proteins are depicted as illustrations.

The docking between tyrosinase and CYBA was successful with significant scores (Eshape score: −402.1 kcal/mol; Eforce score: −457.0 kcal/mol; total score: −859.1 kcal/mol), as shown in Figure 7. The docking scores for FHL2, RBM9, and CYBA were all similar, suggesting that these three binding proteins have similar affinities with respect to tyrosinase binding. When searching for binding residues on the surface of tyrosinase facing toward CYBA, we detected LEU58, PHE60, TYR73, LYS74, ALA75, GLY76, ILE172, ASP173, PRO174, SER175, PHE176, LYS177, PRO178, TYR179, GLY180, ASP181, PHE182, ALA183, THR184, TRP185, VAL189, ASN195, ARG196, ARG197, ILE236, SER237, ASP238, ASP250, ASP251, HIS253, VAL254, MET255, GLY257, TYR258, LYS260, ILE261, GLU262, GLY263, HIS264, MET265, ASP266, HIS267, PRO268, PHE269, PHE270, ARG306, ASP307, GLY308, and THR309. For CYBA, the binding residues were predicted as LYS6, GLN7, PRO8, PRO9, SER10, ASN11, PRO12, PRO13, PRO14, ARG15, PRO16, PRO17, ALA18, GLU19, ALA20, ARG21, LYS22, and LYS23. Comparing the results of Figures 5 to 7, we identified common tyrosinase-binding residues for FHL2, RBM9, and CYBA, namely, LEU58, PHE60, PRO174, SER175, PHE176, LYS177, PRO178, TYR179, GLY180, ASP181, PHE182, ALA183, TRP185, ILE236, SER237, ASP238, GLU262, ASP266, HIS267, PRO268, PHE269, PHE270, ARG306, ASP307, and GLY308. These results suggest that the three proteins share a common binding site with tyrosinase as well as docking behaviors. All binding residues described above were obtained within 5 Å of each protein.

In this study, we identified three binding proteins that interact with tyrosinase, with binding sites near the active site of tyrosinase where the two Cu^2+^ ions are located. Since these two Cu^2+^ ions are necessary for the catalytic activity of tyrosinase toward substrates such as L-tyrosine and L-DOPA, tyrosinase activity could be regulated by FHL2, RBM9, and CYBA. However, this supposition should be confirmed by future studies employing biochemical analyses. With respect to the flexible nature of the active site, Matoba et al. [36] recently suggested that the active tyrosinase center formed by dinuclear Cu^2+^ is flexible during catalysis. Our data suggests modulation of tyrosinase activity via the binding of protein partners. Especially, as these proteins dock near the flexible active site of tyrosinase, conformational changes at the active site after binding could be directly related to substrate accessibility. Therefore, FHL2, RBM9, and CYBA could downregulate the activity of human tyrosinase that might be directly related to the reduction of pigmentation production.
